# High-Precision Extrinsic Calibration for Multi-LiDAR Systems with Narrow FoV via Synergistic Planar and Circular Features

**DOI:** 10.3390/s25206432

**Published:** 2025-10-17

**Authors:** Xinbao Sun, Zhi Zhang, Shuo Xu, Jinyue Liu

**Affiliations:** 1 School of Mechanical Engineering, Hebei University of Technology, Tianjin 300401, China; hebhssxb@163.com (X.S.); x284213945@163.com (S.X.); 2 College of Electrical Engineering, Hebei University of Science and Technology, Shijiazhuang 050000, China; zhangzhi202606@163.com

**Keywords:** extrinsic calibration, multi-LiDAR system, narrow field of view, calibration target detection, nonlinear least squares

## Abstract

Precise extrinsic calibration is a fundamental prerequisite for data fusion in multi-LiDAR systems. However, conventional methods are often encumbered by dependencies on initial estimates, auxiliary sensors, or manual feature selection, which renders them complex, time-consuming, and limited in adaptability across diverse environments. To address these limitations, this paper proposes a novel, high-precision extrinsic calibration method for multi-LiDAR systems with a narrow Field of View (FoV), achieved through the synergistic use of circular and planar features. Our approach commences with the automatic segmentation of the calibration target’s point cloud using an improved VoxelNet. Subsequently, a denoising step, combining RANSAC and a Gaussian Mean Intensity Filter (GMIF), is applied to ensure high-quality feature extraction. From the refined point cloud, planar and circular features are robustly extracted via Principal Component Analysis (PCA) and least-squares fitting, respectively. Finally, the extrinsic parameters are optimized by minimizing a nonlinear objective function formulated with joint constraints from both geometric features. Simulation results validate the high precision of our method, with rotational and translational errors contained within 0.08° and 0.8 cm. Furthermore, real-world experiments confirm its effectiveness and superiority, outperforming conventional point-cloud registration techniques.

## 1. Introduction

In recent years, rapid advancements in laser technology have facilitated the emergence of solid-state LiDAR. Due to its inherent advantages—such as high reliability, lightweight design, and low cost—it is gradually replacing conventional mechanical LiDAR as a key component in environmental perception systems for autonomous vehicles [1]. However, a single solid-state LiDAR is typically limited by a narrow field of view (FoV), which hinders the wide-area coverage required by mobile platforms such as Automated Guided Vehicles (AGVs) and Unmanned Aerial Vehicles (UAVs). Furthermore, in geometrically sparse environments—such as long corridors, tunnels, and open fields—single-LiDAR systems are prone to feature degradation [2,3], thereby undermining the robustness of perception algorithms. Consequently, deploying a multi-LiDAR perception system has become a mainstream solution for achieving a wider perceptual field and richer environmental features [4]. However, the efficacy of this approach critically depends on accurate and robust extrinsic calibration among individual sensors, which remains the main technical challenge.

### 1.1. Motivation

Accurate extrinsic calibration is a fundamental prerequisite for the effective fusion of data from multiple solid-state LiDAR sensors with narrow FoV [5]. Although multi-LiDAR calibration techniques have seen rapid progress in recent years, significant challenges remain in achieving high levels of automation, ensuring robustness under diverse environmental conditions, and improving operational efficiency. Addressing these challenges is the core objective of this study. Specifically, lack of automation poses a major obstacle, some existing methods [6] require manual selection or segmentation of geometric primitives (e.g., planes, corners, or edges) from raw point clouds. This manual process is not only labor-intensive and time-consuming but also limits the scalability and deployment speed of calibration systems in large-scale applications such as autonomous driving. Furthermore, dependence on priors and motion constraints presents another critical challenge, since other approaches [7,8] rely on auxiliary sensors such as IMUs, wheel odometry, or GNSS to provide initial pose estimates, or require the LiDAR platform to follow specific motion trajectories that sufficiently excite the sensor system. Consequently, these methods tightly couple calibration performance with external conditions and the quality of prior data. As a result, large initial pose errors, insufficient movement, or operation in static environments can easily cause such methods to converge to local optima or fail entirely, thereby compromising their robustness and applicability in real-world scenarios.

In light of these limitations, there is a pressing need for a high-precision, fully automated extrinsic calibration method for solid-state LiDAR systems with narrow FoV—one that does not rely on specific motion patterns or auxiliary sensors and remains robust under complex environmental conditions. This study addresses this gap by proposing a novel calibration framework tailored to such requirements.

### 1.2. Contribution

To address the aforementioned challenges, this study proposes an extrinsic calibration method for multiple solid-state LiDARs with narrow fields of view, leveraging a customized calibration board. The primary innovations and contributions of this work can be summarized as follows:

- We propose an automatic method for calibration board detection and segmentation using an improved VoxelNet, which ensures efficient and robust extraction of the board’s point cloud even in complex environments.
- We develop a planar point cloud filtering technique using the GMIF to effectively suppress noise, thereby significantly enhancing the quality of subsequent feature extraction.
- We design a nonlinear optimization framework that jointly constrains planar and circular features. This framework incorporates an innovative adaptive weighting model to balance the contributions of different geometric primitives, leading to substantially improved calibration accuracy.

## 2. Related Work

The rapid advancement of autonomous driving and robotic perception has significantly increased the demand for high-precision and reliable multi-sensor fusion systems. This trend has fostered extensive research on extrinsic calibration methods for multi-LiDAR systems. In this section, we provide a categorized review of existing approaches.

### 2.1. Motion-Based Methods

Motion-based calibration methods, also known as hand–eye calibration [9], originated in robotics. These methods were first used to estimate the extrinsic parameters between a robotic manipulator (“hand”) and a camera (“eye”). The core principle is to estimate the rigid-body transformation between sensors by using observations from different poses. This technique has since been extended to multi-sensor systems, including both multi-camera [10,11] and multi-LiDAR [12,13,14,15] applications. Huang et al. [10] proposed a general hand–eye calibration method based on the Gauss-Helmert model. This model unifies the motion constraints of multiple sensors into a single mathematical framework. Their method simultaneously estimates extrinsic parameters and corrects pose measurement errors. Consequently, it reconstructs accurate sensor motion trajectories, even in high-noise conditions. Schneider et al. [11] used the difference in relative poses from two independent sensor odometries as calibration observations. They performed recursive estimation of the extrinsic parameters within an Unscented Kalman Filter (UKF) framework [12]. This approach improved robustness compared to static batch optimization strategies. Taylor et al. [13] developed a probabilistic calibration method that jointly estimates extrinsics and temporal offsets, providing outlier suppression and uncertainty quantification.

### 2.2. Feature-Based Methods

Feature-based methods estimate extrinsic parameters by extracting geometric features, such as planes, spheres, and corners, from overlapping regions of point clouds. These features are then used to formulate constraint equations. This process is analogous to point cloud registration, where calibration accuracy heavily relies on robust and precise feature extraction. Based on the feature source, these methods are broadly categorized as either artificial target-based or natural structure-based. On one hand, artificial target-based features have shown promising results. For instance, Kim et al. [16] proposed a plane-based method that segments calibration boards from a range image representation, wherein they introduced a novel target completion mechanism to remove outliers and recover inliers on the target surface, which improved feature reliability. Similarly, Zhang et al. [6] developed a method for long-baseline multi-LiDAR systems using spherical targets. The key advantage of spheres is the viewpoint invariance of their projected centers, whereby the method extracts the 3D center of each sphere and uses these centers as stable corresponding points to build geometric constraints. As a result, this approach achieves high accuracy, particularly for systems with large baselines and small FoV overlap. On the other hand, natural structure-based features offer alternative solutions. Specifically, Lai et al. [17] presented a robust method for heterogeneous multi-LiDAR systems, where their approach uses a Gaussian Mixture Model (GMM) to cluster matching points and dynamically adjusts GMM residual weights during optimization to mitigate the impact of outliers. Meanwhile, Lee et al. [18] proposed a planar-object-based strategy that operates without active targets, requiring only at least three linearly independent surface normals in the environment. To further enhance planar feature extraction, Nie et al. [19] introduced an adaptive surface normal estimation technique that accounts for both edge information and non-uniform point distributions. Additionally, Shi et al. [20] proposed an improved dual-LiDAR calibration method using planar features, and notably, they also performed the first systematic uncertainty analysis for dual-LiDAR calibration results.

### 2.3. SLAM-Based Methods

Simultaneous Localization and Mapping (SLAM)-based approaches typically treat extrinsic parameters as state variables and estimate them jointly with sensor poses and map features within a tightly coupled framework. These methods exploit the spatiotemporal consistency across multi-sensor measurements and solve for the optimal extrinsic parameters through various optimization techniques, such as pose graph optimization and factor graph optimization. Liu et al. [21] tackled the extrinsic calibration problem in scenarios with limited or no field-of-view overlap. They generated shared features actively and modeled the constraints between each LiDAR’s pose and the extrinsic parameters using a factor graph framework. Lin et al. [22] adopted a constant velocity motion model and an extended Kalman filter (EKF) to estimate LiDAR extrinsic parameters online. Their method aligns LiDAR measurements with the system’s geometric center during motion. Jiao et al. [23] presented M-LOAM, a multi-LiDAR system that performs extrinsic calibration, real-time odometry, and mapping simultaneously. Their approach emphasizes system integration and efficiency. Zhang et al. [24] utilized geometric features from LiDAR point clouds to build a factor graph. This enabled real-time extrinsic calibration between two LiDAR sensors. Wang et al. [25] proposed a feature fusion strategy based on feature smoothness and spatial distribution. This method enhances feature quality and improves system performance in real time. Cao et al. [26] introduced a fine calibration method based on pose graph optimization. By matching submaps, their approach estimates relative transformations between LiDARs while reducing the effect of odometry drift. Chang et al. [27] developed a motion-excitation-aware filter using a sliding window strategy. The method constructs optimal inter-sensor motion constraints and balances motion distortion with spatial excitation, improving calibration robustness.

To better emphasize the contributions of this study, we systematically compared the proposed method with several recent state-of-the-art calibration methods across key dimensions. A summary of this comparison is provided in Table 1.

In contrast, the method proposed in this paper introduces a calibration target that integrates both planar and circular geometric features. It enables fully automatic and highly robust calibration of narrow FoV multi-LiDAR systems without requiring auxiliary sensors, sensor motion, or high-quality initial estimates. These capabilities represent the core contributions of this work and address a critical gap in existing methods for this specific yet important scenario.

## 3. Methodology

**Figure 1:**
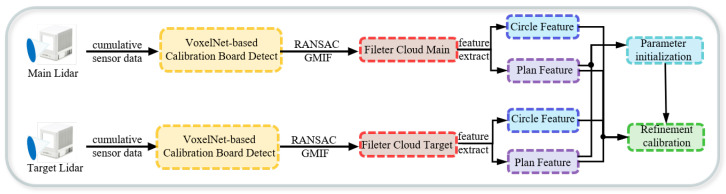
Overview of the proposed multi-LiDAR calibration system. The system comprises three main components: (1) automatic identification and precise segmentation of calibration boards using an improved VoxelNet; (2) extraction of planar and circular geometric features; and (3) joint nonlinear optimization based on multi-feature constraints.

The overall framework of the proposed calibration system is illustrated in Figure 1. The core pipeline comprises three key modules: automatic calibration target recognition, accurate feature extraction, and coupled nonlinear optimization.

The process begins with the automatic recognition and instance segmentation of the calibration target, which is performed using an enhanced VoxelNet algorithm. To ensure the accuracy of the final calibration result, outliers and noise are subsequently removed using the RANSAC algorithm and a GMIF, respectively. The denoised high-quality point cloud is then used to accurately extract planar surfaces and circular center features. In the nonlinear optimization stage, we devise a two-step strategy. First, a reliable initial estimate of the calibration parameters is computed by exploiting correspondences between planar normal vectors. Then, a joint cost function is constructed to refine this initial estimate globally. This function simultaneously incorporates both planar and circular geometric features. This leads to a high-precision estimation of the extrinsic calibration parameters.

### 3.1. Notation

Before presenting the proposed method, we define the notations and conventions used in this paper. The system consists of two solid-state LiDAR sensors. Their coordinate frames are denoted as the primary LiDAR frame $L_{M}$ and the secondary LiDAR frame $L_{S}$. The primary frame $L_{M}$ is used as the reference for calibration. The rigid-body transformation from the secondary frame $L_{S}$ to the primary frame $L_{M}$ is denoted as $T_{S}^{M}\in \mathrm{SE}\left(3\right)$. It consists of a rotation matrix $R_{S}^{M}\in \mathrm{SO}\left(3\right)$ and a translation vector $t_{S}^{M}\in R^{3}$:

(1) $$T_{S}^{M}=\left[\begin{array}{cc} R_{S}^{M} & t_{S}^{M}\\ 0 & 1 \end{array}\right]\in \mathrm{SE}\left(3\right)$$

where SE(3) denotes the special Euclidean group representing 3D rigid-body transformations, SO(3) denotes the special orthogonal group of 3D rotations, and $R^{3}$ represents the three-dimensional Euclidean space.

Since the 3D rotation group SO(3) is locally diffeomorphic to its tangent space at the identity, it allows for a minimal local parameterization. Therefore, we adopt a rotation vector $\varphi \in R^{3}$ as a minimal representation of rotation, which facilitates subsequent nonlinear optimization. The mapping between the rotation vector φ and the corresponding rotation matrix R is established via the exponential and logarithmic maps, defined as follows:

(2) $$\left\{\begin{array}{c} R=\exp({\left(\varphi \right)}^{\wedge })\in \mathrm{SO}\left(3\right)\\ \varphi ={\left(\log\left(R\right)\right)}^{\vee }\in R^{3} \end{array}\right.$$

here, ${\left(\varphi \right)}^{\wedge }$ denotes the skew-symmetric matrix of the vector φ, and the exponential map ${\left(\varphi \right)}^{\wedge }$ can be efficiently computed using Rodrigues’ rotation formula.

For ease of subsequent derivations and explanations, we introduce the following notations:

(3) $$\left\{\begin{array}{c} \mathrm{Exp}(\varphi )=R\\ \mathrm{Log}(R)=\varphi \end{array}\right.$$

### 3.2. Target Detection and Extraction

**Figure 2:**
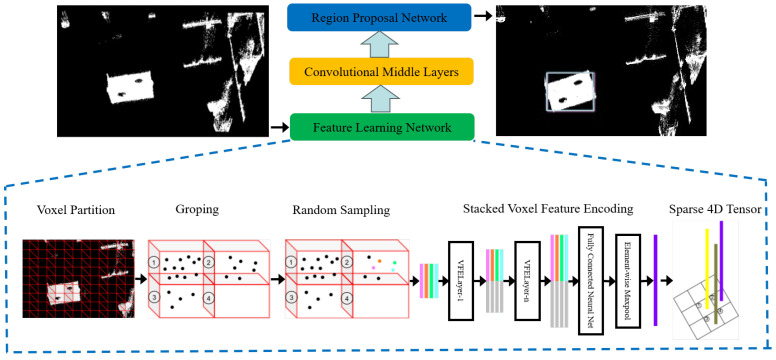
Architecture of the VoxelNet framework. The feature learning network first partitions raw point clouds into voxels, aggregating the points within each voxel into representative feature vectors, resulting in a sparse 4D tensor. This tensor is then passed through intermediate 3D convolutional layers to extract spatial contextual features. Finally, the Region Proposal Network (RPN) generates 3D object proposals for downstream detection tasks.

VoxelNet [28] is a prominent end-to-end deep learning architecture designed for 3D point cloud processing. The architecture consists of three primary components: a feature learning network, convolutional middle layers, and a region proposal network (RPN). In this work, we modify the convolutional middle layers to enhance the network’s ability to identify and extract geometric features. This improvement is particularly beneficial when dealing with calibration target point clouds under complex environmental conditions. The modified VoxelNet architecture is illustrated in Figure 2.

The first step in our processing pipeline involves voxelizing the raw point clouds acquired from the LiDARs. This process involves partitioning the 3D space into a grid of voxels and grouping the points accordingly. Since the point clouds from the two LiDARs often contain millions of points, a downsampling strategy is employed. This step improves both processing efficiency and target detection accuracy. If the number of points within a voxel exceeds a predefined threshold *S*, *S* points are randomly sampled from it. For a voxel containing s<S points, the point set is represented as follows:

(4) $$V={\left\{P_{q}\left(x_{q},y_{q},z_{q},I_{q}\right)\in R^{4}\right\}}_{q=1\ldots s}$$

each point consists of four attributes (x,y,z,I), where *x*, *y*, and *z* denote spatial coordinates and *I* denotes intensity.

Next, we compute the offset of each point in the voxel relative to the point cloud centroid. The resulting offset-augmented point set is denoted as $V_{in}$, and is expressed as follows:

(5) $$V_{in}={\left\{P_{q}^{\prime }\left(x_{q},y_{q},z_{q},I_{q},x_{q}-x_{c},y_{q}-y_{c},z_{q}-z_{c}\right)\in R^{7}\right\}}_{q=1\ldots s}$$

The input $P_{q}^{\prime }$ is processed by a fully convolutional network (FCN) [29], which transforms primitive inputs (e.g., coordinates and intensity) into a learnable high-dimensional feature vector $e_{q}\in R^{m}$, which facilitates subsequent spatial aggregation. Max pooling is applied to $e_{q}$ to obtain the local aggregated feature $e^{\prime }\in R^{m}$ corresponding to *V*. Point-wise concatenation of the features of $e_{q}$ and $e^{\prime }$ yields the output multi-dimensional feature vector $f_{i}^{out}=\left[e_{i},e^{\prime }\right]\in R^{2m}$. The same encoding is applied to all non-empty features to obtain the output feature set:

(6) $$V_{out}={\left\{f_{q}^{out}\right\}}_{q=1\ldots s}$$

Multiple Voxel Feature Encoding (VFE) layers are used to transform the input feature dimension $c_{in}$ into the output feature dimension $c_{out}$. The weight matrix of the linear layer has a shape of $c_{in}\times \left(c_{out}/2\right)$, and the concatenation of intermediate features produces an output with feature dimension $c_{out}$. The m-dimensional convolution operator is represented as $ConvMD(c_{in},c_{out},k,s,p)$. In this formulation, *k* denotes the kernel size, *s* denotes the stride, and *p* denotes the m-dimensional vector specifying per-dimension kernel sizes.

Each intermediate block consists of a 3D convolution, followed by a Rectified Linear Unit (ReLU) and a Batch Normalization (BN) layer. In this work, we adjust the convolutional parameters to better fit the geometric structure of the calibration board in complex scenes. The output feature maps from these layers are passed to a Region Proposal Network (RPN) [30]. The outputs from each module are then upsampled to a common spatial resolution and concatenated into a single high-resolution feature map. This fused representation is mapped to a classification score map and a regression map. The region with the highest confidence score is finally selected as the predicted location of the calibration board.

### 3.3. Plane and Circle Feature Extraction

To obtain a rich and diverse set of co-visible planar and circular features across the multi-LiDAR system, a dynamic data acquisition strategy is used. During calibration, the target is placed at *n* different poses, each visible to all sensors at the same time. This setup provides enough geometric constraints for the following optimization process.

#### 3.3.1. Plane Feature Extraction

Due to the low single-frame point cloud density of non-repetitive scanning solid-state LiDARs, we synchronously collect data from LiDAR sensors $L_{M}$ and $L_{S}$ for τ seconds at each calibration pose to obtain a complete and reliable point cloud of the calibration board.

**Figure 3:**
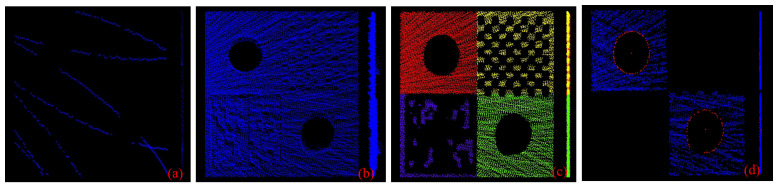
Point cloud processing: (**a**) single-frame point cloud; (**b**) multi-frame accumulated point cloud; (**c**) filtered point cloud after refined segmentation; (**d**) extracted planar and circular features.

Let the aggregated point cloud from $L_{M}$ be represented as $Q_{L_{M}}=\left\{C_{0}^{L_{M}},C_{1}^{L_{M}},\ldots ,C_{n-l}^{L_{M}}\right\}$, and the corresponding dataset from $L_{S}$ as $Q_{L_{S}}=\left\{C_{0}^{L_{S}},C_{1}^{L_{S}},\ldots ,C_{n-l}^{L_{S}}\right\}$. This temporal aggregation improves point cloud density. However, it also introduces cumulative noise. This effect is shown in Figure 3a,b. These subfigures show single-frame and multi-frame aggregated point clouds from frontal and side perspectives, respectively. The accumulated points do not lie on a perfect plane; instead, the side view clearly reveals that they form a point cloud with a noticeable thickness and significant out-of-plane deviations.

To accurately estimate the planar parameters of the calibration board from the point clouds $Q_{L_{M}}$ and $Q_{L_{S}}$, we apply a three-step procedure. This procedure is designed to mitigate the effects of accumulated noise. First, we use the RANSAC algorithm [31] to perform an initial filtering of the point cloud. However, RANSAC may fit a non-target plane due to the board’s imperfect planarity. Such an error would degrade the performance of the subsequent optimization. To address this, we use RANSAC to identify inliers rather than to determine the final plane model. Specifically, we retain only the inlier subset associated with the board’s dominant plane and discard non-target points, such as outliers or p. Second, we perform precise clustering and segmentation on the filtered point cloud using prior knowledge of the board’s geometry. The segmentation result is illustrated in Figure 3c. Finally, the GMIF is applied to further refine the segmented plane points. GMIF computes a geometric mean intensity $I_{M}$ and a threshold σ based on the reflectivity of the points. These metrics are then used to filter out points exhibiting abnormal reflectivity, which often correspond to sensor artifacts or edge points. This process effectively reduces noise and enhances planarity. The resulting point cloud exhibits high fidelity and is well-suited for subsequent accurate plane fitting. The GMIF algorithm is presented in Algorithm 1.  
**Algorithm 1:** Mean Intensity filtering based on Gaussian Newton**Input:** segmented point cloud: $C=\{C_{1},C_{2},\ldots ,C_{N}\}$, $p_{j}\subset C_{k}$, $I_{j}\subset p_{j}$
**Output:**
$C_{\mathrm{GMIF}}=\left\{C_{1}^{\prime },C_{2}^{\prime },\ldots ,C_{N}^{\prime }\right\}$
1:**for **k=1 **to** *N* **do**2: $M_{k}\leftarrow \mathrm{getNumber}\left(C_{k}\right)$3: sum←0.04: **for** j=1 **to** $M_{k}$ **do**5:  $sum\leftarrow sum+I_{j}^{k}$6: **end for**7: $I_{\mathrm{mean}}^{k}\leftarrow sum/M_{k}$8: σ←0.09: **for** j=1 **to** $M_{k}$ **do**10:  $\sigma \leftarrow \sigma +{\left(I_{j}^{k}-I_{\mathrm{mean}}^{k}\right)}^{2}$11: **end for**12: $\sigma^{k}\leftarrow \sqrt{\sigma /M_{k}}$13: $\mathrm{lower}\leftarrow I_{\mathrm{mean}}^{k}-\sigma^{k}$14: $\mathrm{upper}\leftarrow I_{\mathrm{mean}}^{k}+\sigma^{k}$15: **for** j=1 **to** $M_{k}$ **do**16:  **if** $I_{j}^{k}\geq$ lower **and** $I_{j}^{k}\leq \mathrm{upper}$ **then**17:   $C_{k}^{\prime }\leftarrow p_{j}$18:  **end if**19: **end for**20: $C_{\mathrm{GMIF}}\leftarrow C_{k}^{\prime }$21:**end for**22:**return**$C_{\mathrm{GMIF}}$


We further apply Principal Component Analysis (PCA) [32] to the planar point cloud set obtained after GMIF refinement in order to estimate the best-fitting plane. The centroid of the point cloud is denoted by $m_{i}\in R^{3}$, and the unit normal vector of the fitted plane is denoted by $n_{i}\in R^{3}$. Let $X={\left(x,y,z\right)}^{T}$ be an arbitrary inlier point on the plane. The plane equation is then given by

(7) $$n_{i}^{T}\left(X-m_{i}\right)=0$$

#### 3.3.2. Circular Feature Extraction

To accurately extract the 3D circular features of the calibration board, the filtered point cloud is orthogonally projected onto a designated reference plane. Throughout this process, the original 3D coordinates of all points are retained, allowing their projected counterparts to be accurately restored in subsequent steps.

**Figure 4:**
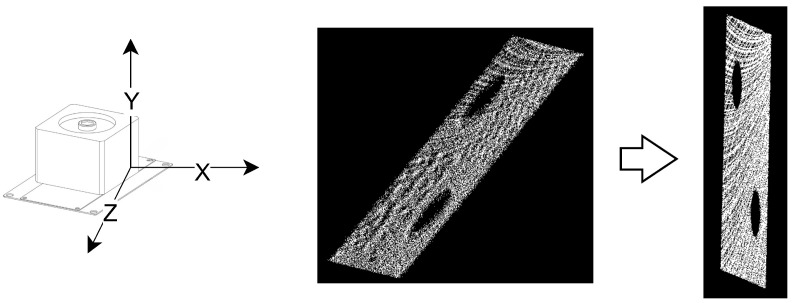
Point cloud geometric projection. The point cloud is orthogonally projected along the positive X-axis onto a plane located at the maximum X-coordinate of all points.

As shown in Figure 4, the reference plane is defined to be perpendicular to the LiDAR’s line of sight, which is aligned with the positive X-axis. To extract the 2D boundary of the projected point set, we apply the Alpha Shapes algorithm [33], which effectively handles both complex and concave geometries. This ensures robust and accurate contour delineation. After boundary extraction, the original 3D coordinates are utilized to recover the X-values of the projected boundary points. This operation lifts the 2D boundary into 3D space, reconstructing a contour point cloud denoted as $C_{bdp}^{\prime }$. A minimal subset of points is randomly sampled from $C_{bdp}^{\prime }$ to construct a candidate 3D circle, characterized by the following equations:

(8) $$\left\{\begin{array}{c} n\cdot (p-p_{0})=0\\ {∥p-c∥}_{2}^{2}=R^{2} \end{array}\right.$$

where n is the normal vector of the plane containing the 3D circle, p is any arbitrary point on the 3D circle, $p_{0}$ is an arbitrary point from the minimal subset, c is the coordinate of the circle’s center, and *R* denotes the radius.

The distances between all points in $C_{bdp}^{\prime }$ and the candidate 3D circles are computed. Points whose distances fall below a predefined threshold are marked. This process is repeated until the maximum number of iterations is reached. The 3D point cloud with the highest number of inlier markings is then selected. The least squares method is then used to refit the 3D point cloud, yielding the optimized 3D circle contour and its corresponding center. The fitted contour points and the circle’s center are shown in Figure 3d.

### 3.4. Nonlinear Optimization

To achieve high-precision calibration, the entire nonlinear optimization process is divided into two stages.

#### 3.4.1. Parameter Initialization

The initial estimation of extrinsic parameters is critical to ensure the convergence and final accuracy of subsequent nonlinear optimization in multi-LiDAR calibration. This section focuses exclusively on the initial estimation of the rotational component of the extrinsic parameters. This is primarily because rotational parameters lie on the Special Orthogonal group SO(3), which forms a nonlinear manifold. This structural property makes the optimization of rotation highly sensitive to its initial value. In contrast, the translation parameters lie in the Euclidean space $R^{3}$, where the optimization is less dependent on the initial guess and generally exhibits better convergence properties. Therefore, this section is dedicated to estimating the initial rotation to establish a solid foundation for the subsequent fine-grained optimization of the full six-degree-of-freedom (6-DoF) extrinsic parameters.

We construct the constraints by matching the normal vectors of the same calibration board plane captured from different viewpoints by the LiDARs. Let $n_{i}^{L_{M}}$ and $n_{i}^{L_{S}}$ denote the normal vectors of the calibration board plane extracted at the *i*-th pose from LiDARs $L_{M}$ and $L_{S}$, respectively. Accordingly, the residual is defined as the angle between the corresponding plane normal vectors from the two LiDARs. The cost function is formulated as follows:

(9) $$L\left(R_{L_{S}}^{L_{M}}\right)=\sum_{i=1}^{N}∥e_{i}^{R}{∥}^{2}=\sum_{i=1}^{N}{∥\left(R_{L_{S}}^{L_{M}}n_{i}^{L_{S}}\right)\times n_{i}^{L_{M}}∥}^{2}$$

During the optimization process, the rotational parameters are iteratively updated to minimize the angular residual between the corresponding plane normal vectors. As a result, the transformed normal vector gradually aligns with the target normal vector. The entire initialization process can be represented as follows:

(10) $${\hat{\varphi }}_{L_{S}}^{L_{M}*}=\underset{R_{L_{S}}^{L_{M}}\in \mathrm{SO}\left(3\right)}{arg min}L\;\left(R_{L_{S}}^{L_{M}}\right)$$

#### 3.4.2. Refined Calibration

**Figure 5:**
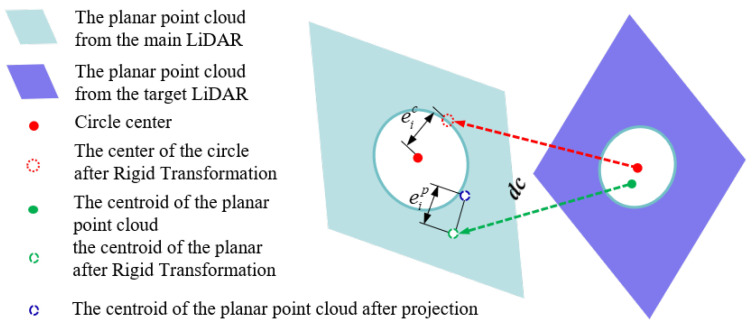
Joint feature constraints in the calibration process. Planar and circular geometric constraints are jointly formulated as part of the optimization objective function. These constraints guide the iterative minimization of residuals for accurate estimation of inter-LiDAR extrinsic parameters.

Building on the initial rotation parameters obtained in Section 3.4.1, this section aims to refine the full extrinsic parameters between multiple LiDARs. To achieve this, a multi-constrained coupled optimization model is constructed by jointly calibrating both planar and circular features of the calibration board. This approach enhances the accuracy and robustness of the resulting calibration. The feature constraint relationships are shown in Figure 5.

(1) Centroid-to-Plane Constraint

Unlike Section 3.4.1, which formulates constraints based solely on minimizing the angular discrepancy between corresponding plane normals, this section introduces the Euclidean distance from the calibration board’s centroid to the matched plane as a primary constraint term. This formulation ensures compatibility with the subsequent circle constraint, which is likewise defined in terms of Euclidean distance.

The residual term for the Euclidean distance between the centroid $m_{i}^{L_{S}}$ and the plane $\Pi_{i}^{L_{M}}$ is formulated as follows:

(11) $$\left\{\begin{array}{c} {\hat{m}}_{i}^{L_{S}}=R_{L_{S}}^{L_{M}}m_{i}^{L_{S}}+t_{L_{S}}^{L_{M}}\\ e_{i}^{p}\left(R_{L_{S}}^{L_{M}},t_{L_{S}}^{L_{M}}\right)={∥{\left(n_{i}^{L_{M}}\right)}^{T}\left({\hat{m}}_{i}^{L_{S}}-m_{i}^{L_{M}}\right)∥}_{2} \end{array}\right.$$

(2) Center-to-Center Constraint

The constraint on circular features is defined as the Euclidean distance residual between corresponding circle centers. To mitigate the influence of potential circular fitting errors and to balance the relative contributions of the planar and circular constraints within the objective function, we introduce a weighting factor for the circular constraint:

(12) $$\omega_{j}=\left\{\begin{array}{cc} 1 & \mathrm{if}\;|\xi_{j}|\leq \delta \\ \frac{\delta }{|\xi_{j}|} & \mathrm{if}\;|\xi_{j}|>\delta \end{array}\right.$$

where $\xi_{j}=r_{j}^{\mathrm{true}}-r_{j}^{\mathrm{est}}$, $r_{j}^{\mathrm{true}}$ and $r_{j}^{\mathrm{est}}$ represent the true and estimated radii of the circular feature, respectively, and δ is a predefined threshold.

The residual term for the distance between $c_{j}^{L_{M}}$ and $c_{j}^{L_{S}}$ is defined as follows:

(13) $$e_{j}^{c}\left(R_{L_{S}}^{L_{M}},t_{L_{S}}^{L_{M}}\right)=\omega_{j}{∥\left({\hat{c}}_{j}^{L_{S}}-c_{j}^{L_{M}}\right)∥}_{2}$$

herein, ${\hat{c}}_{j}^{L_{S}}=R_{L_{S}}^{L_{M}}c_{j}^{L_{S}}+t_{L_{S}}^{L_{M}}$.

(3) Iterative Optimization

During the refined optimization stage, the cost function is defined as follows:

(14) $$L\left(R_{L_{S}}^{L_{M}},t_{L_{S}}^{L_{M}}\right)=\sum_{i=1}^{m}∥e_{i}^{p}{∥}^{2}+\sum_{j=1}^{n}{∥e_{j}^{c}∥}^{2}$$

The refined calibration is formulated as the following optimization problem:

(15) $$\left(\varphi_{L_{S}}^{{L_{M}}^{*}},t_{L_{S}}^{{L_{M}}^{*}}\right)=\underset{R_{L_{S}}^{L_{M}}\in \mathrm{SO}\left(3\right),t_{L_{S}}^{L_{M}}\in R^{3}}{arg min}L\left(R_{L_{S}}^{L_{M}},t_{L_{S}}^{L_{M}}\right)$$

## 4. Experiments

To comprehensively evaluate the accuracy and robustness of the proposed algorithm, we conducted extensive experiments using both simulated and real-world datasets.

### 4.1. Implementation Details

To evaluate the proposed algorithm, simulations and real-world experiments were conducted using two Livox Mid-40 solid-state LiDAR sensors. These sensors were manufactured by DJI Technology Co., Ltd., located in Shenzhen, China. Each sensor has a circular field of view (FoV) of 38.4°, an angular accuracy of 0.05°, and a sampling frequency of 10 Hz. A customized calibration board with geometric features was used as the target. The board consisted of four square regions, each with a side length of 0.6 m. Circular holes with a diameter of 0.3 m were cut into the top-left and bottom-right regions. These two regions were primarily used during calibration, as they contained both the planar and circular features required by our method. The training platform was a desktop computer running Ubuntu 20.04, equipped with an NVIDIA Quadro A6000 GPU (32 GB VRAM). The deployment platform was an NVIDIA Jetson Orin Nano, also running Ubuntu 20.04. A custom-developed mobile robot served as the experimental platform. All programs were implemented in C++. The Point Cloud Library (PCL) was used for preprocessing, while Eigen and Sophus were employed for matrix operations. Nonlinear optimization was performed using the Ceres Solver.

### 4.2. Simulated Experiment

**Figure 6:**
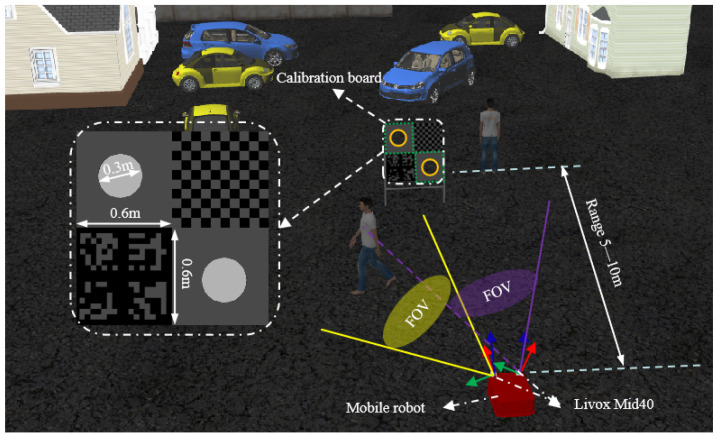
Validation in the simulated environment.

Gazebo was used to generate simulated data for our experiments. As an advanced open-source simulator, Gazebo is capable of simulating various types of sensor data, including that from the Livox Mid-40 solid-state LiDAR used in this study. To evaluate the accuracy and robustness of the proposed algorithm, a complex simulation environment featuring people, vehicles, a calibration board, and buildings was set up, as illustrated in Figure 6. Gaussian noise was added to the simulated point cloud data to better mimic LiDAR behavior in real-world scenarios. Specifically, zero-mean Gaussian noise with a standard deviation of 0.01 m was applied to each point to simulate the LiDAR’s scanning behavior under realistic conditions.

Given that the Livox Mid-40 has a limited field of view (FoV) of only 38.4°, the roll and pitch angles between the two LiDAR sensors were restricted to ±10°, while the yaw angle was constrained to ±15. This constraint ensures sufficient overlap between the point clouds of the two sensors, enabling reliable acquisition of calibration data. The distance between the calibration board and the mobile robot varied throughout the process. Experimental observations indicated that maintaining a distance between 5 and 10 m led to more uniform and sufficient point cloud coverage on the calibration board surface.

Properly selecting the number of calibration board point cloud samples is crucial for accurate estimation of the calibration parameters. An excessively large sample size significantly increases data collection time. In contrast, too few samples may reduce calibration accuracy. To explore this trade-off, we quantitatively evaluate the relationship between sample size and calibration accuracy using simulation experiments. The specific procedure is as follows:

- Define the ground-truth calibration parameters.
- Collect a dataset of 100 calibration board point cloud instances.
- Generate subsets of varying sizes via random sampling without replacement.

**Figure 7:**
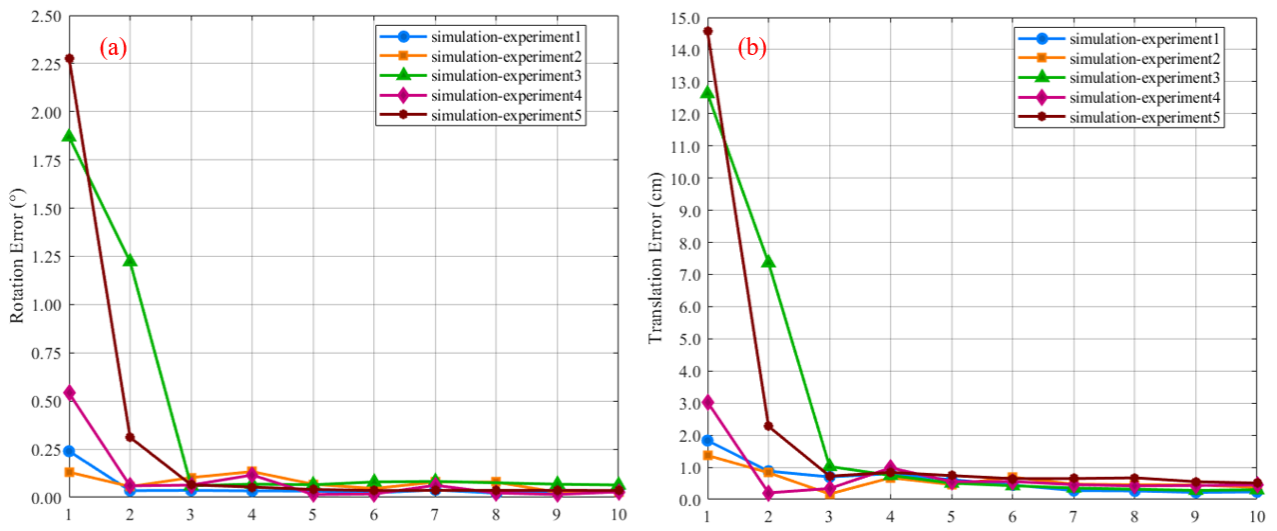
Impact of the number of calibration boards on the accuracy of extrinsic calibration: (**a**) variation in rotational calibration error with respect to the number of calibration boards; (**b**) variation in translational calibration error with respect to the number of calibration boards.

The relative pose between the two LiDARs is known in the simulation environment. This allows calibration accuracy to be evaluated in a rigorous and quantitative manner. To this end, we define the ground-truth transformation as $T_{\mathrm{true}}=\left\{R_{\mathrm{true}},t_{\mathrm{true}}\right\}$. The corresponding estimated transformation is denoted as $T_{\mathrm{est}}=\left\{R_{\mathrm{est}},t_{\mathrm{est}}\right\}$. Based on this, we define the following error metrics:

(16) $$\left\{\begin{array}{c} \varepsilon_{R}={∥\mathrm{Log}\left(R_{\mathrm{true}}^{⊤}R_{\mathrm{est}}\right)∥}_{2}\\ \varepsilon_{t}={∥t_{\mathrm{true}}-t_{\mathrm{est}}∥}_{2} \end{array}\right.$$

here, $\varepsilon_{R}$ and $\varepsilon_{t}$ denote the rotational and translational errors, respectively. Figure 7 quantitatively illustrates the variation in calibration error with respect to the number of calibration board samples across five independent experiments. Figure 7a,b illustrate the trends of rotation and translation calibration errors as a function of the number of randomly selected calibration patterns, respectively. In both plots, the horizontal axis represents the number of samples, and the vertical axis indicates the error between the estimated values and the ground truth. The experimental results demonstrate that the system achieves convergence when the sample size, *n*, is greater than 5 (*n* > 5).

**Figure 8:**
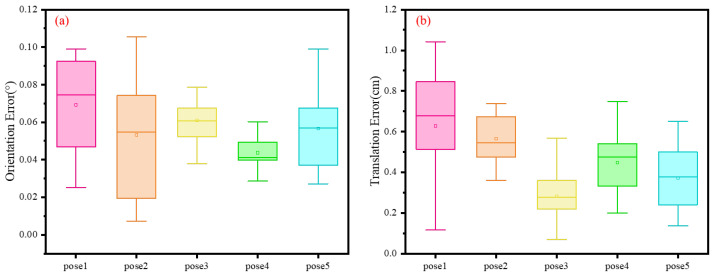
Distribution of errors between the estimated calibration results and the ground truth: (**a**) distribution of rotation errors; (**b**) distribution of translation errors.

To evaluate the robustness and generalization capability of our calibration algorithm, we conducted simulation experiments using five distinct ground-truth configurations. These configurations were further used to investigate the influence of varying ground-truth parameters on algorithm performance. Each parameter set comprises five calibration board point cloud samples. For each set, we conducted 10 repeated trials under identical initial conditions to reduce the influence of random factors. This enhances the stability and reproducibility of the experimental results. The five sets of ground-truth parameters, along with their corresponding Euler angle representations, are summarized in Table 2. Table 3 presents the number of independent experiments conducted under different pose conditions. It also lists the rotation and translation errors of the estimated calibration results with respect to the ground truth. For a more intuitive visualization of these simulation results, the error distributions for rotation and translation are plotted in Figure 8. Specifically, Figure 8a,b illustrate the distribution of rotation and translation errors across different ground truth poses, respectively. In these plots, the horizontal axis represents the various ground truth poses, while the vertical axis indicates the error between the estimated results and their corresponding ground truth.

**Figure 9:**
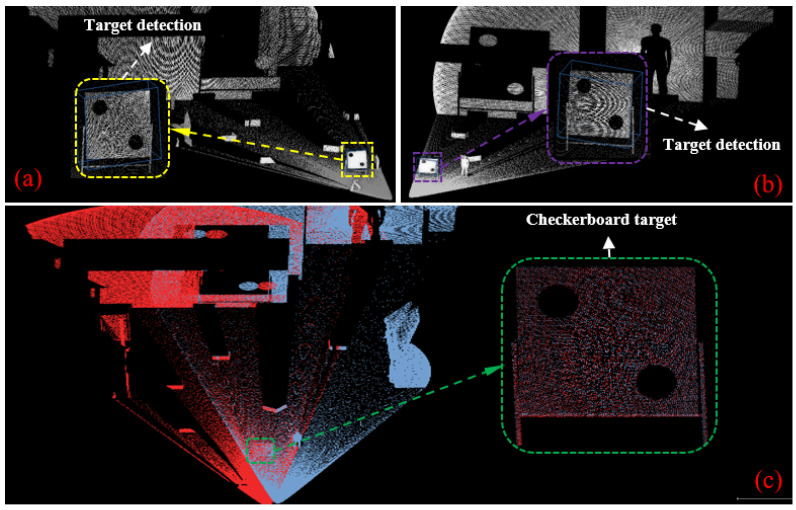
LiDAR point cloud registration using target detection in a simulated environment: (**a**) detection of the calibration pattern in the primary LiDAR’s point cloud; (**b**) detection of the calibration pattern in the target LiDAR’s point cloud; (**c**) registration result of the point clouds from the primary and target LiDARs.

Figure 9 illustrates the registration results demonstrating the alignment between object detection outputs and LiDAR point clouds in a simulated environment. Specifically, Figure 9a,b show the calibration board detected by the primary LiDAR and the target LiDAR, respectively. Figure 9c presents the final point cloud registration result obtained from both LiDARs.

The simulation results show that across all five experimental sets, the median rotational error remained below 0.08°, and the median translational error stayed below 0.8 cm. This indicates that the overall calibration error was effectively controlled. These results confirm that the proposed algorithm achieves consistently high calibration accuracy across varying parameter settings.

### 4.3. Real-World Experiments

**Figure 10:**
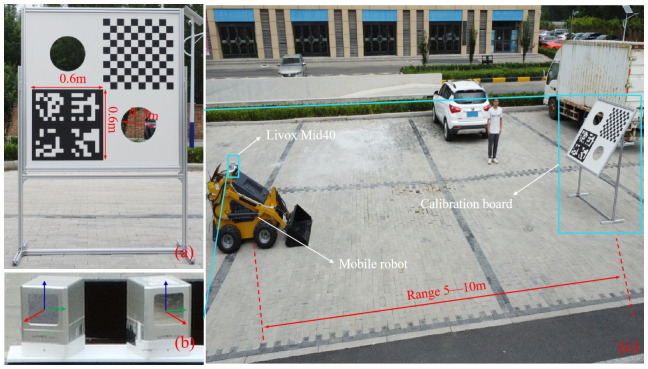
Setup and results of the real-world experiments: (**a**) calibration board; (**b**) installed LiDAR sensors; (**c**) configured multi-LiDAR experimental environment.

**Figure 11:**
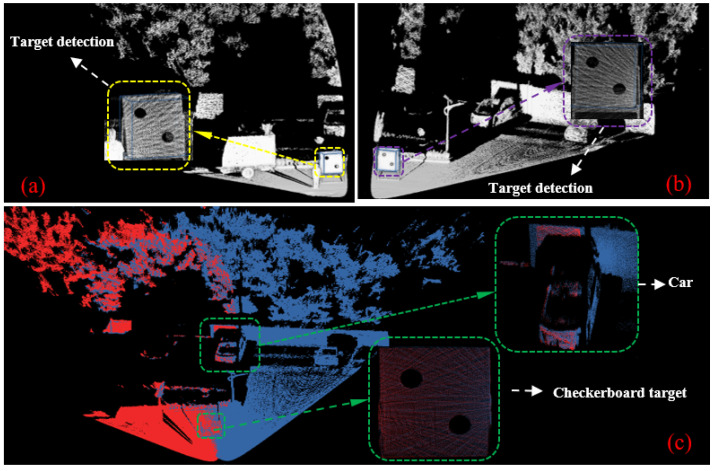
Performance of object detection and point cloud registration in realistic: (**a**) detection of the calibration pattern in the primary LiDAR’s point cloud; (**b**) detection of the calibration pattern in the target LiDAR’s point cloud; (**c**) registration result of the point clouds from the primary and target LiDARs.

Figure 10 illustrates the experimental setup and real-world environment. As shown in Figure 10a, the calibration board used in the real-world experiments has the same dimensions and shape as the one used in simulation. It is suspended in the air by a metal support frame. Figure 10b shows two Livox Mid-40 LiDAR sensors mounted on the chassis of a mobile robot. The sensors are secured using custom 3D-printed connectors. Figure 10c presents the actual outdoor test environment. During data acquisition, the procedure was similar to that in the simulation. The calibration board was placed sequentially at distances ranging from 5 to 10 m from the mobile robot. For each position, both LiDAR sensors simultaneously collected data for 10 s. Figure 11 shows the results of target detection and point cloud registration in the real-world setting. Figure 11a,b display the calibration board detected by the primary and target LiDARs, respectively. Figure 11c shows the final registration result of the point clouds from both sensors.

**Figure 12:**
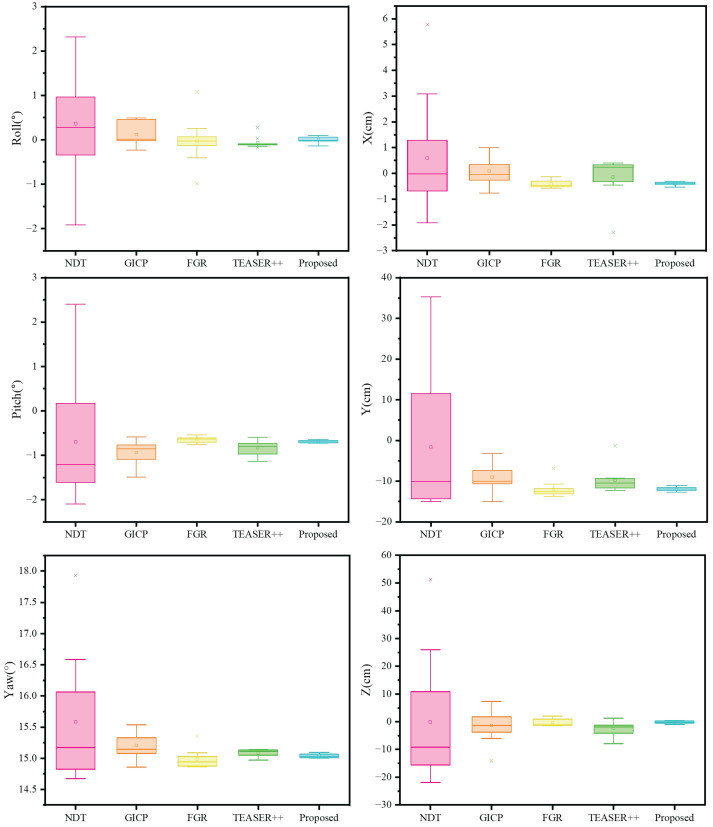
Field evaluation using Livox Mid-40 LiDARs. The figure compares the proposed method with NDT, GICP, FGR, and TEASER++. Each box summarizes the results over 10 independent runs.

To evaluate the performance of the proposed calibration method, we benchmarked it against four recent algorithms: NDT [34], GICP [35], FGR [36], and TEASER++ [37]. To ensure a fair and rigorous comparison, the raw point cloud data for all methods underwent preprocessing using a voxel grid filter with a leaf size of 0.05 m. Furthermore, each method was subjected to 10 independent trials to ensure statistical robustness. Table 4 provides a quantitative comparison of the evaluated methods. It includes the average execution time, as well as the median and standard deviation of the estimated extrinsic parameters across all six degrees of freedom (6-DoF). For a more intuitive visualization of the results, Figure 12 shows the distribution of the rotational (roll, pitch, yaw) and translational (x, y, z) components for each method.

According to Table 4 and Figure 12, NDT is the fastest method. However, its large standard deviation makes it unsuitable for high-precision tasks. GICP is also computationally efficient and more accurate than NDT. Nevertheless, compared to the state-of-the-art methods FGR and TEASER++, GICP shows less consistency, indicated by a wider box plot. In contrast, both FGR and TEASER++ deliver highly consistent results. Their small standard deviations and narrow box plots reflect excellent precision. However, both methods suffer from occasional failures, which are visible as prominent outliers in their error distributions. Notably, our proposed method achieves a computational time comparable to FGR’s. More importantly, it outperforms all four benchmark algorithms by achieving the lowest median error and superior consistency. Furthermore, our method is highly robust, showing no significant outliers across any of the experimental trials.

Overall, the box plot results demonstrate that the proposed method outperforms the baseline methods in both accuracy and robustness. It provides more reliable LiDAR-to-LiDAR extrinsic calibration.

## 5. Conclusions and Future Work

This paper proposes an automatic and high-precision calibration method for multiple narrow FoV LiDARs. The method automatically extracts and segments the calibration board point cloud using an improved VoxelNet-based neural network. RANSAC and GMIF filters are applied to remove noise, thereby improving plane extraction accuracy. During optimization, a weighting strategy is introduced to reduce the influence of noise in circular features. Multiple simulation and real-world experiments were conducted to validate the effectiveness of the proposed calibration method. In simulation experiments, the estimated results were compared with ground-truth data, demonstrating a rotation error below $0.08^{\circ }$ and a translation error under 0.8 cm. In real-world experiments, the proposed method was compared with NDT, GICP, FGR, and TEASER++ and consistently outperformed these baselines in both accuracy and robustness. These findings demonstrate that the proposed method achieves high calibration accuracy and strong robustness in both simulated and real-world scenarios.

Despite the promising performance of our method in the current experiments, we also acknowledge its limitations. The real-world experiments in this paper were primarily conducted in relatively static and structured environments. This setup provided a clear baseline for validating the core performance of our proposed framework. However, real-world applications such as autonomous driving and robotic navigation often involve dynamic and highly cluttered scenes. Interference and severe occlusion from dynamic objects, such as pedestrians and moving vehicles, undoubtedly pose new challenges to the stable extraction and matching of features. To address these challenges and further enhance the practical utility of our method, we plan to pursue the following research in our future work. First, we will conduct supplementary experiments in dynamic and cluttered scenarios to comprehensively evaluate and optimize the generalization ability and robustness of our method. Second, we will incorporate advanced 2D or 3D segmentation networks to achieve precise extraction of target features from complex backgrounds. Finally, another important direction for future research is to extend our calibration framework to the joint calibration of heterogeneous sensors such as LiDAR and cameras.

## Figures and Tables

**Table 1:** Comparison of different calibration methods.

Method	Key Approaches	Auxiliary Sensor Dependency	Automation Level	Robustness Under FoV Limitations	Drawbacks	Main Contribution
Motion-based	Multimodal And Temporal Calibration [13]	LiDAR + GNSS + camera	Fully automatic	Medium	Depends on high-quality motion data	Probabilistic and fully automatic calibration without initialization
	Observability-aware Calibration [15]	LiDAR + GNSS + IMU	Fully automatic	Medium	Not applicable in indoor environments	Online and continuous optimization
Feature-based	Heterogeneous LiDAR Calibration [17]	LiDAR	Requires manual initialization	Medium	Depends on well-structured scenes	Handles calibration between heterogeneous LiDARs
	Adaptive Surface Normal Calibration [19]	LiDAR	Requires manual initialization	High	Not applicable in indoor environments	Robust calibration in sparse and poorly structured point clouds
SLAM-based	Pose Graph Calibration [26]	LiDAR	Fully automatic	Low	Unsuitable for high-speed or degenerate scenarios	Achieves globally consistent calibration
	Versatile Self-Calibration [27]	LiDAR	Fully automatic	Low	Unsuitable for high-speed or degenerate scenarios	Pose graph-based optimization for robust and consistent calibration
Ours	-	LiDAR	Fully automatic	High	Requires a dedicated calibration target	Circle-plane joint optimization enhances calibration in challenging environments

**Table 2:** Extrinsic parameter configurations.

LiDAR Position	Roll (°)	Pitch (°)	Yaw (°)	X (cm)	Y (cm)	Z (cm)
pose1	0	0	0	0	40	0
pose2	0	0	-5	0	40	0
pose3	4	−4	−4	0	40	0
pose4	4	−4	−10	0	40	0
pose5	8	−8	−12	0	40	0

**Table 3:** Performance evaluation under varying ground truth configurations.

LiDAR Position	No. of Trials	Orientation Error (°) [Median±SD]	Translation Error (cm) [Median±SD]
pose1	10	0.07458 ± 0.02487	0.67825 ± 0.31342
pose2	10	0.05483 ± 0.03298	0.54427 ± 0.12831
pose3	10	0.06078 ± 0.01236	0.27693 ± 0.1427
pose4	10	0.04111 ± 0.00934	0.47408 ± 0.15487
pose5	10	0.05693 ± 0.02088	0.37655 ± 0.17177

Median: middle value of trials; SD: standard deviation.

**Table 4:** Comparison of different registration methods.

Method	NDT	GICP	FGR	TEASER++	Proposed
No. of Trials	10	10	10	10	10
Avg. Time (s)	1.193	7.002	10.701	15.606	10.062
Roll (°) [Median ± SD]	0.280 ± 1.184	0.012 ± 0.273	−0.026 ± 0.514	−0.101 ± 0.131	−0.011 ± 0.074
Pitch (°) [Median ± SD]	−1.212 ± 1.431	−0.857 ± 0.264	−0.631 ± 0.070	−0.804 ± 0.164	−0.697 ± 0.033
Yaw (°) [Median ± SD]	15.174 ± 1.037	15.147 ± 0.222	14.943 ± 0.149	15.108 ± 0.058	15.030 ± 0.033
X (°) [Median ± SD]	−0.019 ± 2.314	−0.050 ± 0.578	−0.470 ± 0.159	−0.446 ± 1.123	−0.386 ± 0.070
Y (°) [Median ± SD]	−10.076 ± 16.836	−10.056 ± 3.778	−12.588 ± 2.005	−10.493 ± 3.141	−11.878 ± 0.466
Z (°) [Median ± SD]	−9.207 ± 23.233	−1.336 ± 6.255	−1.045 ± 1.207	−1.830 ± 2.700	−0.195 ± 0.468

Median: middle value of trials; SD: standard deviation.

## Data Availability

The data are not publicly available.
